# The CalculAuthor: determining authorship using a simple-to-use, fair, objective, and transparent process

**DOI:** 10.1186/s13104-023-06597-4

**Published:** 2023-11-12

**Authors:** Russell Seth Martins, Mohsin Ali Mustafa, Asad Saulat Fatimi, Nosheen Nasir, Alina Pervez, Sarah Nadeem

**Affiliations:** 1 Division of Thoracic Surgery, Department of Surgery, JFK University Medical Center, Hackensack Meridian School of Medicine, Hackensack Meridian Health (HMH) Network, Edison, NJ 08820 USA; 2 CITRIC Center for Clinical Best Practices, Aga Khan University, Karachi, 74800 Pakistan; 3 Medical College, Aga Khan University, Karachi, 74800 Pakistan; 4 Department of Medicine, Aga Khan University, Karachi, 74800 Pakistan

**Keywords:** Authorship, Academic research, Taxonomy

## Abstract

Authorship determination on a research article remains a largely subjective process. Existing guidelines on authorship taxonomy lack objectivity and are more useful in determining who deserves authorship rather than determining the order of authors. To promote best practices in authorship taxonomy, we developed an authorship rubric that provides a fair, objective, and transparent means of crediting authorship. We christened this tool the “CalculAuthor”. The following steps are to be undertaken to create a scoring system based on the requirements of the projects: determining creditable criteria, assigning credit weightages, deciding levels of contribution, determining each author’s contribution, calculating authorship scores and ranking. These must be performed by or in close collaboration with the primary investigator (PI), with conflicts being resolved at the PI’s discretion. All team members should be informed about the authorship determination process early in the project and their agreement regarding its use must be obtained. While the CalculAuthor was developed to be used in medical research, its customizability enables it to be employed in any field of academia. We recommend that the CalculAuthor be piloted within institutions before its mainstream adoption, and any institution-specific factors should be considered to make the process more efficient and suitable.

**Supplementary Information:**

The online version contains supplementary material available at 10.1186/s13104-023-06597-4.

## Introduction

While the conclusion of a research project is accompanied by feelings of accomplishment stemming from the culmination of one’s hard work, there is also an expectation of being rewarded for one’s efforts with a fair authorship position. However, the grim reality of many research teams across the world is one where authorship determination remains a largely subjective process [1]. Authorship conventions vary across academic disciplines, countries, institutions, and even amongst research groups within the same discipline [2]. These nuances across disciplines are captured by the authorship guidelines published by relevant bodies within different disciplines (e.g., the International Committee of Medical Journal Editors in the biomedical sciences, the American Sociological Association in the social sciences, and the American Physical Society in physics) [3]. Some fields, primarily economics, employ alphabetic sequencing when listing authors [4]. However, this practice also leads to problematic repercussions. This norm gives an unfair advantage to researchers with last name initials that are early in the alphabet. Moreover, this “alphabetical discrimination” makes researchers wary of who they collaborate with, so as to have a higher authorship rank [5, 6]. We have captured some of these subtleties amongst the STEM (science, technology, engineering, and mathematics) fields in Table 1.

**Table 1:** Connotations of authorship in the various STEM fields (adapted from a community discussion on academia stack exchange [20])

Field	Connotation
Math, computer science, and related fields [21]	• Lists authors on papers in alphabetical order [4] • All authors are assumed to have contributed equally and are listed alphabetically •Authors reject the notion that their contributions can be strictly ordered
Physics and engineering	• A first author is usually the lead student or worker on the particular project from which the paper originates • If there are multiple contributors on a particular project, first authorship is usually awarded to whoever has done the most work in preparing the manuscript for publication • If contributions are truly deemed equivalent, first authorships may be shared among different authors to recognize comparable contributions throughout the combined work • The last author is often a senior academician, who may have advised or directed the first author but not have performed any of the experimentation themselves • Different conventions exist even for the various subfields of physics
Biology, chemistry, and medicine	• The first author is usually the individual who puts the most labor into the work • The last author is usually the primary investigator, though this practice is relatively recent. Previously, the primary investigator would be listed as the first author • If contributions are truly deemed equivalent, first authorships may be shared among different authors to recognize comparable contributions throughout the combined work • First author is considered the most important authorship position • Middle authors are sequenced in order of decreasing contributions to the various components of the manuscript (data acquisition, data analysis, and manuscript writing) • Last author denotes the role of seniority, mentor, thought leader, or subject expert • First authorship is the basis of application for awards, prizes, or fellowships

Some key considerations for authorship designation were identified by Marušić et al. namely the proper definition of authorship criteria, implications of authorship sequence, and authorship practices in collaborative research projects [7]. Guidelines on authorship taxonomy in the biomedical sciences have been published by the International Committee of Medical Journal Editors (ICMJE) [8] and Contributor Roles Taxonomy (CRediT) [9]. Holcombe et al. also developed *tenzing*, a web application and R package that can help facilitate reporting of contributorship information in manuscripts and journal articles [10]. However, the ICMJE and CRediT frameworks lack objectivity and are more useful in determining whose contributions warrant an authorship rather than determining the order of authors. Holcombe et al. note the lack of degree of contributorship as a key limitation of CRediT and by extension, *tenzing *[10]. Moreover, the ICMJE criteria have been criticized as being unduly restrictive, harsh, and difficult to realistically follow [11, 12]. However, improper adherence to objective authorship criteria may give rise to unethical academic practices such as ghost authorship and guest authorship. Often, academic hierarchy and institutional seniority supersede actual contributions, with the existing system rarely being challenged. Not being suitably compensated inevitably leads to feelings of frustration, demotivation, and a distaste towards medical research as a whole [1]. The Committee on Publication Ethics (COPE) has outlined several suggestions for authors to negotiate terms of authorship and resolve authorship issues [3]. It has also stressed the responsibility of institutions and journals to recognize suboptimal authorship practices [3]. Furthermore, an approach described by Tscharntke et al. to address author contribution challenges involves explicitly indicating methods used to determine authorship, which can help avoid conflicts and increase satisfaction amongst authors [13]. However, given the ubiquity of this issue in the realm of academia, there is an urgent need to explore improvements to the existing system.

The Center for Clinical Best Practices (CCBP) at the Aga Khan University (AKU) in Pakistan is tasked with the standardization of clinical care and academic standards at AKU. In order to promote best practices in authorship taxonomy for CCBP and other institutional research projects, the CCBP team created and piloted an authorship rubric that provides a fair, objective, and transparent means of crediting authorship. In this commentary, we describe the process of development of this innovative authorship calculation algorithm, christened the “CalculAuthor”.

### Approach and outcomes

Our algorithm outlines the following steps that are to be followed sequentially for each individual research project, as the creditable criteria, criteria weightages, and levels of contribution are expected to differ from project to project. These must be performed by or in close collaboration with the PI. Ideally, all team members should be informed about the authorship determination process before the commencement of a study, and their general agreement regarding its use must be obtained.

1. *Determining Creditable Criteria for Authorship*: A list of criteria, encompassing all the aspects of the research project. These criteria may be founded upon those provided by the ICMJE [8] and CRediT [9], with criteria being modified, added, or deleted as deemed necessary with respect to a particular research project. It is advisable to request the entire research team to review the list of criteria, so as to ensure that no creditable criteria have been overlooked. Moreover, a greater degree of specificity in determining creditable criteria ensures a more comprehensive process of determining the level of contribution being made with respect to the overall project for individuals working on similar aspects of the project. In other words, it is recommended that broader domains, such as “manuscript writing”, be further subdivided into well-defined tasks to ensure that appropriate credit is given for different responsibilities under the same overall domain of the project.
2. *Assigning Credit Weightages to Criteria*: Weightages amounting to a total of 100 points must be attributed to each criterion. This can be achieved by first scoring each criterion in a range of 1 (least weightage) to 10 (most weightage) points, keeping in mind the relative effort and expertise required for tasks included in each criterion. These scores can then be scaled to a total of 100 points. This scaling up to 100 points is important to achieve mathematical unity, which will dissuade from retrospective changes to the weightages of certain creditable criteria. The attribution of weightages to each of the creditable criteria should be done by the primary investigator and any other team members closely involved with project supervision and oversight, preferably at the beginning of the project. Once weightages have been assigned to each criterion, it is preferable to request the entire research team to review and provide their general agreement regarding the weightages. If, at the end of the project, any of the authors feel that the weightages of the creditable criteria warrant rethinking, the PI may choose to modify weightages and re-solicit the authors’ agreement with the new weightages.
3. *Levels of Contribution*: Levels of contribution must be decided as the most appropriate for a specific project, with each level securing a fixed percentage of the total possible points available for each criterion. At our institution, we have successfully used Major (100% of points i.e., a multiplication factor = 1), Minor/Moderate (50% of points; multiplication factor = 0.5), and No Contribution (0% of points; multiplication factor = 0). Other iterations, such as Major (100%), Moderate (50%), Minor (25%), and No (0%) Contribution could be considered. The degree of involvement that constitutes (and differentiates) specific levels of contribution is entirely dependent on the nature of each creditable criteria (e.g. number of patient records collected during data acquisition), and should thus be decided on by the team collectively at the start of a project.
4. *Determining Each Author’s Contribution*: Each author must be asked to independently categorize their level contribution for each criterion. To promote transparency, this self-scoring should take place on an online spreadsheet, such as Google Sheets or Microsoft Excel Online, shared to each author’s email. Online spreadsheets additionally possess a useful feature whereby changes made by the authors can be tracked. The PI must then review each author’s self-reported contributions for accuracy. Conflicts regarding authors’ contributions across criteria can be resolved through discussion with the PI.
5. *Calculating Authorship Scores*: An author’s authorship points for each criterion must be calculated by multiplying the total available points for each criterion by the multiplication factor of each level of contribution. Total authorship points (/100) can be obtained by adding the authorship points across each criterion. The calculation process can be easily automated using Microsoft Excel or Google Sheets formulae.
6. *Creating Authorship Ranking*: The total authorship points calculated in the previous step are arranged in descending order to obtain an authorship ranking. In the event of tied rankings due to an equal number of points, the order of authorship for the concerned authors can be left to the PI’s discretion after they have judiciously and holistically evaluated the contributions of the tied authors to the project. As per convention, the PI, if senior author, may opt to be placed at the end of the authorship list. The final authorship ranking should be reviewed by all the authors in the research team. Dissatisfaction on the part of any author(s) may be resolved through discussion with the PI. Agreement on the final authorship ranking must be recorded for all authors, preferably with their signatures.

Table 2 shows the results of the authorship determination process using the CalculAuthor for the present article.

**Table 2:** Results of the authorship determination process using the CalculAuthor

Creditable criteria	Weightages	RSM	MAM	ASF	NN	AP	SN*
CalculAuthor conceptualization	40	Major	Major	Minor	Minor	No	Major
Manuscript conceptualization	20	Major	Major	No	No	No	Minor
Writing (introduction)	10	Major	No	Major	No	Minor	No
Writing (process)	14	Major	No	No	No	No	No
Writing (comment)	10	Major	No	Minor	No	Major	No
Critical review	6	Minor	Major	Minor	Major	Minor	Major
Overall	100	97	66	38	26	18	56

Major Contribution = 100% of available points; Moderate/Minor Contribution = 50% of available points; No Contribution = 0% of available points

^*^The senior-most author opted to be placed at the end of the author list

### Piloting experience

Our team successfully piloted the CalculAuthor on 10 different research papers (including the current article) which had a total of 128 authors. Of these, 2 papers have been published in a peer-reviewed medical journal [14, 15]. Amongst these 128 authors, 22 (17.2%) were Assistant Professors, 3 (2.3%) were Associate Professors, and 17 (13.3%) were Professors. Encouragingly, 61 (47.7%) of authors were students/trainees/research associates. The remaining 25 authors (19.5%) were other clinical or research investigators. The first author position was occupied by a student/trainee/research associate in 9/10 (90%) of research papers. Moreover, 24/30 (80%) of the top three authorship positions across the 10 research papers were occupied by students/trainees/research associates. At the initiation of each project, the PI disclosed the future use of the CalculAuthor for the purposes of authorship determination and explained how each component of the CalculAuthor methodology operated. This initial debriefing took place over a virtual meeting. For each project, creditable criteria and their weightages were determined through consensus before the start of the project. All queries and concerns regarding the CalculAuthor methodology were clarified at the start of the project, as well as later on during the course of the project if any concerns arose. For the most part, the introduction of the CalculAuthor was met with general approval by project members and was viewed especially positively by junior members of the project. The only objections that arose were related to the assignment of relative weightages to the creditable criteria. However, these were resolved following group discussions, and the eventual consensuses were accepted by all authors. We received two key suggestions during the preliminary stages of the development of the CalculAuthor. Firstly, that the attribution of creditable contributions for each of the authors be performed by the PI or other project lead/supervisor, so as to minimize the additional workflow and avoid inflated self-reports of contributions. However, we chose not to incorporate this element of feedback and instead retain the self-reporting framework, as this would allow authors to be more satisfied with their eventual placement. The transparent nature of self-reporting contribution, and the PI-mediated conflict resolution mechanism, was expected to limit and resolve issues related to inflated self-reported contributions. Secondly, in the preliminary version of the CalculAuthor, the weightages for each creditable criteria were decided by group consensus at the start of the project. However, this approach resulted in frequent disagreements regarding the assigned weightages, and was time-consuming, complicated, and frequently unproductive. We received a suggestion that the allocation of weightages to each creditable criteria should be performed by the PI and project lead/supervisor themselves, with the agreement of other authors being sought after the allocation. We chose to incorporate this element of feedback and observed it to result in a much more time efficient and streamlined workflow. All authors were responsible for self-recording their contributions on an Excel workbook to which all team members had access. The PIs were responsible for regularly checking the shared workbook for accuracy of reported contributions. In general, all authors were in agreement regarding the fairness of the rankings, the transparency and objectivity of the rubric in determining authorship positions, and the weightages assigned to contributions assigned to each aspect of the research project in deciding authorship rankings. Unfortunately, we did not use any objective methods (e.g. a survey) to quantify satisfaction, agreement/disagreement, or other objections to the CalculAuthor. However, it was extremely heartening to see students, trainees, and research associates (i.e. the juniormost members of the research teams) occupying top positions in the authorship list. The automated CalculAuthor tool (Microsoft Excel spreadsheet) used for the present article is shown in Additional File 1.

### Outlook

The CalculAuthor promotes consistency and objectivity in the authorship ranking process by quantifying effort across component tasks. The transparency of the process deems it fair, with a right to appeal to the PI in cases of conflict or dissatisfaction. The customizability in selecting criteria enables its application to all types of research. A noteworthy point of the CalculAuthor is that it operates under the premise that any degree of contribution towards any of the creditable criteria warrants recognition as an author. In addition, having predetermined definitions for the level of contribution to each creditable criteria also negates biases during authors’ self-reporting of their contributions (e.g. it is fairly common for authors to overestimate their contributions to a project [16]). Although efforts have been made previously to rank authors using a rubric [17], they have not considered quantifying the extent of contribution made by individuals for each criterion. This addition is particularly useful in large projects, where multiple authors may play a part in a single component task. We have outlined some existing authorship rubrics and highlighted how they differ from the CalculAuthor in Table 3. Of note, the majority of existing tools have been developed prior to the development of the CRediT taxonomy in 2015. Thus, they lack the flexibility to account for the contributorship roles described in CRediT, due to which their relevance to modern-day authorship determination is restricted. A recent review by Whetstone et al. in 2020 presented a critique of existing authorship rubrics. They concluded that a major limitation of these rubrics was their restriction of creditable criteria to only traditional roles in a project, and their inflexibility to account for contributorship in more unconventional areas such as programming and software design (which may be key aspects of contemporary projects) [18].

**Table 3:** What makes the CalculAuthor different from existing authorship rubrics*?

Rubric	Year of Publication	Discipline	Key Differentiating Features from the CalculAuthor
Analytical hierarchy process model (AHPM) [22]	2006	Engineering	• Weighting of creditable criteria is bound by subjective interpretable terminology such as ‘Criteria 1 contributes “weakly more/strongly more/demonstrably more/absolutely more” than Criteria 2ʹ • Relatively more complicated to use, understand, or explain to co-authors who are not acquainted with mathematical concepts • Authorship order determined using user-derived ranked fractional contributions • No process described for breaking tied scores or other conflict resolution • Lack of PI oversight with regards to accuracy of self-reporting contributions; necessitates that author be unbiased about their own contributions
Authorship determination scorecard (ADS) and authorship tiebreaker scorecard (ATS) [23, 24]	2014 (Based on Winston et al. [25])	Psychology	• Creditable criteria are non-customizable • Weighting of creditable criteria is non-customizable and restrictive (using assigned fixed-point values) • Levels of contribution are quantified by distributing the points available for a certain authorship criterion between all authors rather than giving each author an independent score for each criterion • Tiebreaker rubric includes categories not provided in the first rubric, such as data entry, writing the abstract, or completing the IRB application; however, no information on what to do if scores on the tiebreaker rubric are also tied • No process described for other conflict resolution • Lack of PI oversight with regards to accuracy of self-reporting contributions; necessitates that author be unbiased about their own contributions
Authorship matrix [26]	2014	Engineering	• Authorship only warranted if individual contributes to at least three of the four rubric categories • Creditable criteria are non-customizable • Tie-breaking by placing junior author ahead of the senior author • No process described for other conflict resolution • Authorship order determined by the descending order of net contribution percentage rather than total score • Lack of PI oversight with regards to accuracy of self-reporting contributions; necessitates that author be unbiased about their own contributions
Authorship scale [27]	1997	Medicine	• Creditable criteria are non-customizable • No weighting of creditable criteria • Levels of contribution for creditable criteria can be variably quantified but are bound by subjective interpretable terminology such as “minimal”, “some”, and “significant” • Suggests some tasks do not warrant authorship but instead acknowledgement (e.g., data collection, providing participants, funding, or administrative support) • Conflicts and disputes are to be resolved by the head of the department (not by the PI) • In the case of a tie for first author, the author with the higher score on “conception” is given preference; when scores are equal, decision is made by consensus of the authors • Breaking tied scores for other authorship positions is the responsibility of the first author. If controversy remains, a committee will resolve the dispute, otherwise the authorship order will be determined by the head of the department
Authorship schema [20]	1985	Psychology	• Levels of contribution are quantified by distributing the points available for a certain authorship criterion between all authors rather than giving each author an independent score for each criterion • Tied scores are broken using a coin toss • No process described for other conflict resolution • Individuals awarded less than 50 points are not awarded authorship and contributions are mentioned in acknowledgements • Points are assigned to authorship criteria by consensus among authors rather than self-reporting by authors
Kosslyn’s criteria [28]	2002	Cognitive Science	• Creditable criteria are non-customizable • Point values assigned to all evaluative criteria sum to 1,000 points to be divided among users; the weightage/values assigned to each criterion can be modified • Contributors awarded more than 0 but less than 10% of the total points do not warrant authorship and are mentioned in the acknowledgements; individuals on the threshold are offered a chance to take on a bigger role to achieve authorship credit • No process described for breaking tied scores or other conflict resolution
Simple framework for evaluating authorial contribution (SFEAC) [29]	2016	Engineering	• Creditable criteria are non-customizable • No weighting of creditable criteria • Levels of contribution for creditable criteria have fixed point values for three thresholds • Levels of contribution for creditable criteria are bound by subjective interpretable terminology such as “minimal”, “significant”, and “major” • A pre-determined total point threshold is set by the PI or by mutual agreement to determine cut-offs for authorship credit • No process described for breaking tied scores or other conflict resolution
Worksheet for authorship [30]	1987	Ecology	• Creditable criteria are non-customizable • No weighting of creditable criteria • A “natural break” at the lower end of contribution scores is used to determine who is awarded authorship credit • All evaluative criteria are assigned 100 points each to be divided among authors rather than independent scores for each criterion • No process described for breaking tied scores or other conflict resolution
Five-step authorship framework [31]	2014	Medicine	• Specific to industry-sponsored clinical trial publications • Provides a framework within which an authorship rubric can be developed for a specific clinical trial, but no pre-specified system to quantify authorship contribution beyond “substantial” • Although creditable criteria can be tailored to a specific clinical trial, only “substantial” contributions will count towards authorship which underplays the role of those who might have made minor contributions in multiple criteria • A committee keeps track of authorship contributions to account for accuracy in self-reporting contributions • Can be used to determine whether a collaborator’s contributions warrant authorship, but no way to rank said contributions against other collaborators and determine authorship order
Survey-weighted analytic hierarchy process (S-AHP) [32]	2021	Medicine	• Can be used to determine whether a collaborator’s contribution warrants authorship as well as authorship rank based on quantification of ICMJE criteria metrics • Authorship only warranted if at least one component from ICMJE criterion 1, and one component from ICMJE criterion 2 has been contributed to, in addition to mandatory contribution to final approval and accountability for the study • Creditable criteria are non-customizable • Levels of contribution for specific creditable criteria are non-customizable • No process described for breaking tied scores or other conflict resolution • Lack of PI oversight with regards to accuracy of self-reporting contributions; necessitates that author be unbiased about their own contributions

*ICMJE* International Committee of Medical Journal Editors, *IRB* Institutional Review Board, *PI* principal investigator

^*^This is a non-exhaustive list of existing authorship rubrics and is intended to highlight only key differentiating features when compared to the CalculAuthor

The objective ranking method levels the playing field for all authors irrespective of seniority. Women have faced more disagreements in authorship naming and ranking compared to their male counterparts and have found it difficult to plead their case [19]. A transparent process will accord the higher authorship positions to key contributors without any dispute, sparing unnecessary and uncomfortable confrontation. This method will also compel the members to play an active role if they want a higher position in the author list. In addition, our method helps to credit every effort made by authors in individual criterion, which is reflected in the final scoring, instead of simply negating minor efforts completely. This small differentiation would also help to decrease the chance of authors obtaining the same score. Furthermore, gift authorships can be dissuaded to some extent owing to the transparency of the process.

Assessing coauthors’ contributions in collaborative scientific work is challenging due to its subjective nature. Biases may arise from self-perceptions, interpersonal interactions, and power dynamics, all of which may influence credit allocation and potentially impact fair recognition. Herz et al. highlighted the necessity of mitigating biases arising from over-estimation of the amount and importance of one's own contributions to a project by promoting transparency in credit allocation [16]. Furthermore, Eggert et al. suggests fair authorship allocation by identifying all contributors, negotiating relative contributions, assigning authorship based on a specified criterion, designating a principal investigator, and disclosing the complete list of contributors in the publication [13]. The CalculAuthor provides a tool whereby all of the above is possible in a practical, feasible, and relatively uncomplicated manner.

To make the process more streamlined, we encourage future authors to introduce authors to the authorship ranking process early on in a research project and incorporate the teams’ feedback to tailor the CalculAuthor to a specific research team or project. This should be followed with an active effort to keep track of the tasks being performed by the individuals, for verification and calculation of final rank at the end. While the CalculAuthor was developed to be used in medical research, its customizability enables it to be employed in any field of academia. We recommend that the CalculAuthor be piloted within institutions before its mainstream adoption, and any institution-specific factors should be considered to make the process more efficient and suitable.

### Supplementary Information

**Additional File 1**. Demonstration of the CalculAuthor tool to determine authorship for the present article.

## Data Availability

Not applicable.

## Abbreviations

PI: Primary investigator
ICMJE: International Committee of Medical Journal Editors
CRediT: Contributor roles taxonomy
CCBP: Center for Clinical Best Practices
AKU: Aga Khan University
